# Tomographic assessment of bone changes in atrophic maxilla treated by split-crest technique and dental implants with platelet-rich fibrin and NanoBone^®^ versus platelet-rich fibrin alone: Randomized controlled trial

**DOI:** 10.1186/s12903-024-04420-5

**Published:** 2024-06-14

**Authors:** Maged Anis, Ahmed Reda Abdelrahman, Rasha Attia, Amr Zahran

**Affiliations:** 1 Department of Periodontology, Faculty of Dentistry, Modern Sciences and Arts University, Giza, Egypt; 2 Department of Periodontology, Faculty of Dentistry, Cairo University, El-Manial, Cairo, 11553 Egypt

**Keywords:** Alveolar ridge augmentation, Ridge splitting, PRF, Piezo-electric surgery, Nanocrystalline hydroxyapatite, Dental implant, Crestal bone changes

## Abstract

**Background:**

This study evaluated the clinical benefits of adding NanoBone^®^ with split-crest technique and simultaneous implant placement covered with platelet-rich fibrin membrane in horizontally deficient maxillary ridges in terms of crestal and horizontal bone changes and patient morbidity.

**Methods:**

Forty patients indicated for maxillary ridge splitting and simultaneous implant placement were assigned randomly to the study groups: control group (Platelet Rich Fibrin membrane) and test group (Platelet Rich Fibrin membrane + Nanobone^®^). The Cone Beam Computed Tomography Fusion technique was utilized to assess crestal and horizontal bone changes after five months of the surgical procedure. Patient morbidity was recorded for one week post-surgical.

**Results:**

Five months post-surgical, buccal crestal bone resorption was 1.26 ± 0.58 mm for the control group and 1.14 ± 0.63 mm for the test group. Lingual crestal bone resorption was 1.40 ± 0.66 mm for the control group and 1.47 ± 0.68 mm for the test group. Horizontal bone width gain was 1.46 ± 0.44 mm for the control group and 1.29 ± 0.73 mm for the test group. There was no significant statistical difference between study groups regarding crestal and horizontal bone changes and patient morbidity.

**Conclusions:**

The tomographic assessment of NanoBone^®^ addition in this study resulted in no statistically significant difference between study groups regarding crestal and horizontal bone changes and patient morbidity. More randomized controlled clinical trials on gap fill comparing different bone grafting materials versus no grafting should be conducted.

**Clinicaltrials.gov registration number:**

NCT02836678, 13^th^ January 2017.

## Background 

Dental implant placement requires sufficient alveolar bone volume to be functionally and esthetically successful. To reconstruct deficient dimensions and provide an ideal bony bed for implant placement, multiple alveolar ridge preservation and augmentation techniques have been developed. Those techniques include socket preservation, block bone grafting, guided bone regeneration, distraction osteogenesis, split-crest technique, or a combined approach from the previous techniques [1–3].

Split-crest procedure for horizontal alveolar ridge augmentation provides many advantages for the indicated cases. It permits simultaneous implant placement in the same surgical procedure which decreases the number of surgeries needed, reduces treatment time and cost, decreases surgical complications, and reduces patient morbidity [4, 5].

Dimensional bone changes after split-crest technique and simultaneous implant placement in terms of horizontal and crestal bone resorption are evident in the literature [6]. To maximize bone gains and minimize bone loss, various materials have been used in conjunction with split-crest technique, such as bone grafts, barrier membranes, platelet-derived concentrates, bone morphogenic proteins, or a combination of them [7–9].

In this context, and to avoid drawbacks related to certain types of bone grafts such as increased cost and patient morbidity associated with autogenous bone graft harvesting and possible risk of infection transmission and antigenicity related to allografts and xenografts, Nanobone^®^ was selected to be utilized in association with split-crest technique in this study.

Nanobone^®^ is bioceramic bone graft material consisting of non-sintered nanocrystalline hydroxyapatite immersed in a silica gel matrix. It is characterized by numerous open bonds, and a porosity range from 60 to 80% leading to an extremely large internal surface area. It has osteoconductive properties and a bone formation rate higher than other hydroxyapatite preparations and it completely resorbs after 8 months of placement [10, 11].

Histological studies on Nanobone^®^ showed complete bone formation and complete graft material resorption in contrast to bioceramics produced by the ordinary method, its osteoconductive and biomimetic properties, and suggested it has osteoinductive characteristics as well [10, 11]. Nanobone^®^ has been used for socket preservation, filling the jumping gap in immediate implant placement, sinus augmentation, and alveolar ridge splitting [12, 13].

Choukroun et al. in 2001 introduced an easy and simple way to produce PRF [14]. PRF is a platelet concentrate that encompasses elements that allow for optimal healing. Growth factors and cytokines released during PRF production (such as vascular endothelial growth factor, insulin-like growth factor-1, transforming growth factor-β1, platelet-derived growth factor α and β, interleukin 1β, interleukin 4, and tumor necrosis factor α) are the elements responsible for PRF clinical effects. These elements improve soft and hard tissue healing by stimulating the production of collagen leading to an increase in wound strength and inducing callus formation [15].

PRF and PRF membranes have been evaluated in the treatment of infrabony defects, guided tissue regeneration, gingival recession, post-extraction healing, and bone regeneration with excellent results. Studies investigated the effect of PRF in intrabony defect treatment either alone as a fill material, as a membrane to induce guided tissue regeneration, or in combination with other grafting materials, showed a greater pocket depth reduction, increased clinical attachment level gain, and more bone fill other than the comparative groups [16–18].

The current study aimed to accurately assess bone changes using the Cone Beam Computed Tomography Fusion (CBCTF) technique after using Nanobone^®^ with platelet-rich fibrin versus platelet-rich fibrin alone in atrophic maxilla treated with split-crest technique and dental implants.

## Materials and methods

### Study design

The current study was designed as a randomized controlled clinical trial and was conducted in the Periodontology Department, Faculty of Dentistry, Cairo University. The study was performed following the consort guidelines and the ethical principles of the Declaration of Helsinki and its later amendments. The Center of Evidence-Based Dentistry-Cairo University and Research Ethics Committee, Faculty of Dentistry, Cairo University reviewed and accepted the study protocol, and was registered on Clinicaltrials.gov database (n∘ NCT02836678).

Signing a written informed consent form was a prerequisite for enrollment in the study to ensure that the patients understood the aim of the study and any potential harm. Patients were encouraged to ask about the study and alternative treatment modalities were also discussed with them.

This trial hypothesis was null hypothesis to prove that there are no differences in buccal and lingual crestal bone changes, horizontal bone changes, and patient morbidity between the test group (PRF membrane + Nanobone^®^) and control group (PRF membrane) in horizontally deficient maxillary ridges treated with split-crest technique and simultaneous implant placement.

### Study population

Included participants were adult patients, with horizontally deficient maxillary alveolar ridges who needed horizontal ridge augmentation and were indicated for split-crest technique and simultaneous implant placement. Participants were subjected to clinical examination evaluating their periodontal and occlusal conditions. Bone dimensions in the areas of interest were measured and recorded using cone beam computed tomography (CBCT).

Inclusion criteria were the following:

- Age: >18 years old.
- Maxillary edentulous Seibert class I defect [19].
- Edentulous ridge width of < 6 mm.
- Good oral hygiene.
- Patients agree to sign the informed consent.

The exclusion criteria were the following:

- Smoking.
- Systemic disease that jeopardize implant installation or surgical procedures.
- Poor oral hygiene.
- History of chemotherapy or radiotherapy.
- Psychological disorders.
- Surgical site pathosis.
- Pregnancy.
- Deficient apico-coronal and\or mesio-distal dimensions for the final restoration.

### Randomization, allocation concealment, and blinding

Two lists were created representing the two study groups. An online random sequence generator^1^ was used to generate a randomization list of two groups in which the number from one to 40 was randomly distributed and equally assigned to both groups. Numbers from one to 40 were written on paper and inserted in similar, opaque, and sealed envelopes. On surgery day, the participant was asked to pick an envelope which was then opened to crossmatch the number within it with the randomization list to identify its group. The randomization list was kept with an investigator not involved in the study. The outcomes measure assessor and the data analyst were blinded.

### Outcomes

#### Buccal and lingual crestal bone changes

They were measured by tomographic evaluation of the implant site via CBCTF. CBCTF was performed by importing CBCT data files performed one week after surgery (T1) and after five months of follow-up (T2) into OnDemand 3D software and merging them producing one file with both images overlaying each other. The two images at the two different time points were overlapped and exactly matched by the software algorithm and presented with different colors for each image. Buccal and lingual crestal bone changes were calculated by measuring the difference between the two overlapping images.

#### Horizontal bone changes

The horizontal bone changes were measured by the same CBCTF technique performed by merging CBCT scan data performed preoperatively (T0) and after five months post-operatively T2. Horizontal bone width Changes were assessed by measuring the difference between the two overlapping images.

#### Pain

It was recorded by the participants using the Numerical Rating scale (NRS) questionnaire. The participants were instructed to record the pain on a scale ranging from 0 “no pain” to 10 “worst pain” first thing in the morning for the first seven days post-operatively. NRS questionnaire was collected at the week one recall appointment.

#### Swelling

It was recorded by the participants using the Visual Rating Scale (VRS) questionnaire and it was collected at week one recall appointment. The participants were instructed to record the presence of the swelling and its degree as follows:

- 1 No swelling.
- 2 Slight (Swelling at the surgical site intraorally that cannot be recognized by others).
- 3 Moderate (Swelling at the surgical site intraorally that can be recognized by others).
- 4 Extensive (Extra-oral swelling that extends beyond the surgical site).

### Sample size

The sample size calculation was performed considering a minimum of one mm clinical difference in bone dimensional change between the control and test groups which would be relevant clinically. Considering 80% power, 5% significance level, and 1.0 as standard deviation, 17 patients in each group were required. To compensate for dropout, the number was increased to 20. G*Power software was used for sample size calculation (University of Düsseldorf, Düsseldorf, Germany).

### Pre-surgical protocol

Phase I therapy including supragingival scaling, subgingival debridement, and oral hygiene instructions was performed before the surgical intervention. Four weeks later, patients were evaluated to ensure their adherence to the oral hygiene instructions.

### Surgical procedures

**Fig. 1:**
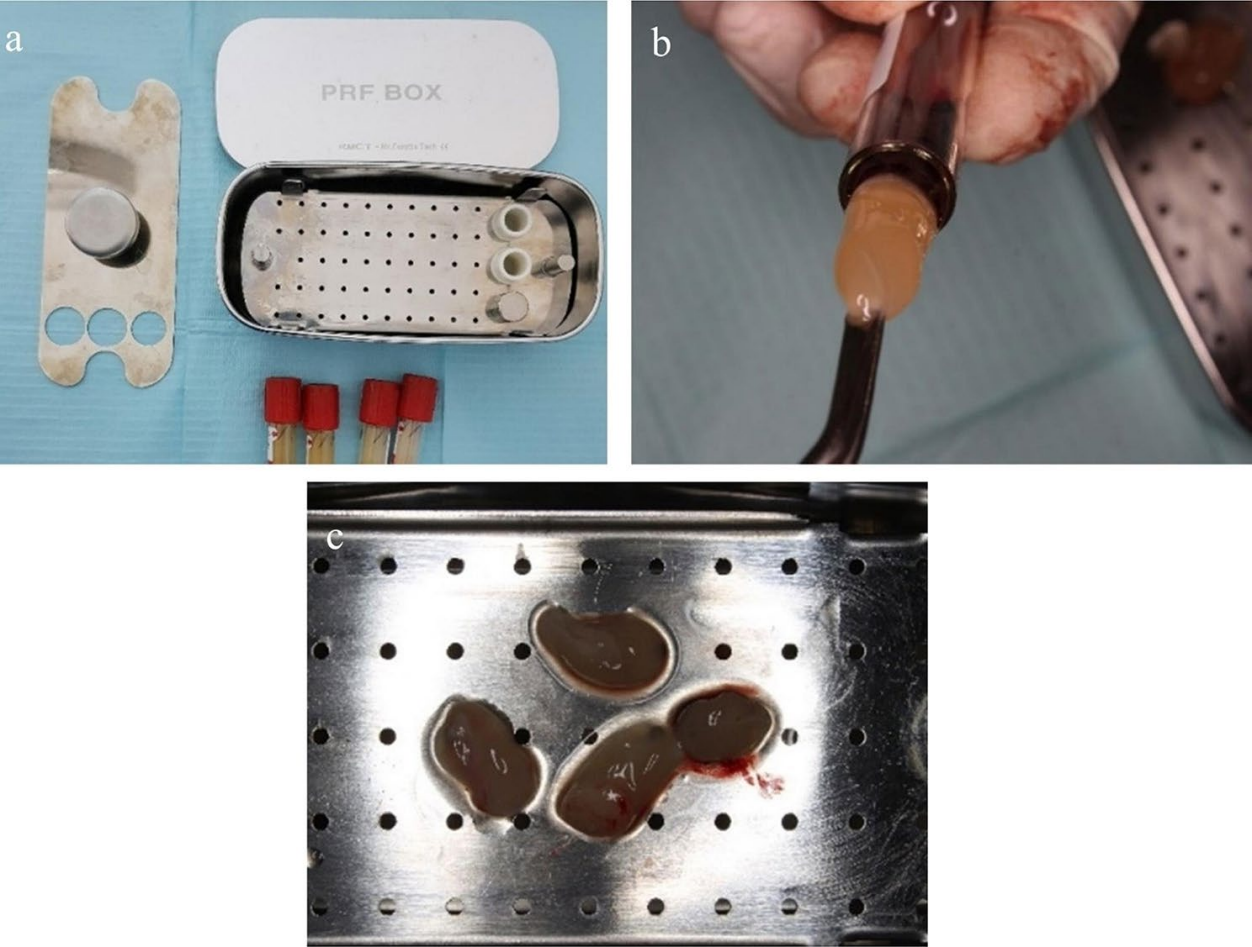
PRF preparation (**a**) PRF box, (**b**) PRF formed after centrifuging, (**c**) PRF placed in the PRF box to form a membrane

Before the surgical intervention and to produce the PRF membrane, blood was collected from the patient in 10 ml plain vacutainers and centrifuged for 10 min at 2,000 rpm using a low-speed lab benchtop centrifuge machine^2^ with a relative centrifugal force of 700 g. The clot containing platelets was collected from the tube and the attached red blood cells were discarded. The PRF clot was transferred to the PRF Box^3^ and compressed to produce the fibrin membrane (Fig. 1).

**Fig. 2:**
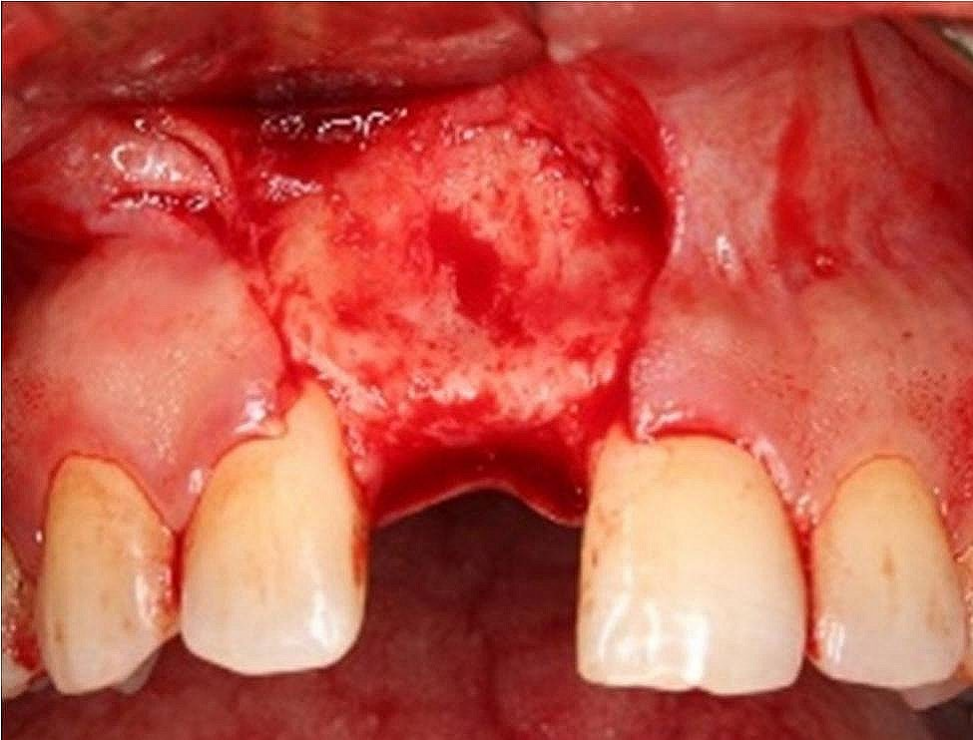
Flap design utilized for surgical exposure; sub-crestal incision with two vertical releasing incisions

All procedures were performed under local anesthesia^4^ using a local infiltration technique. A full mucoperiosteal trapezoidal flap was performed to expose the surgical site including a sub-crestal incision and two vertical releasing incisions performed buccally (Fig. 2).

A piezoelectrical surgical device^5^ was used to perform the osteotomies using the SG1 piezosurgical tip. The first osteotomy was performed at the crest of the ridge along the edentulous span and ending two mm from the nearest neighboring tooth/teeth when present or extended two mm beyond the implantation site when neighboring tooth/teeth were absent.

**Fig. 3:**
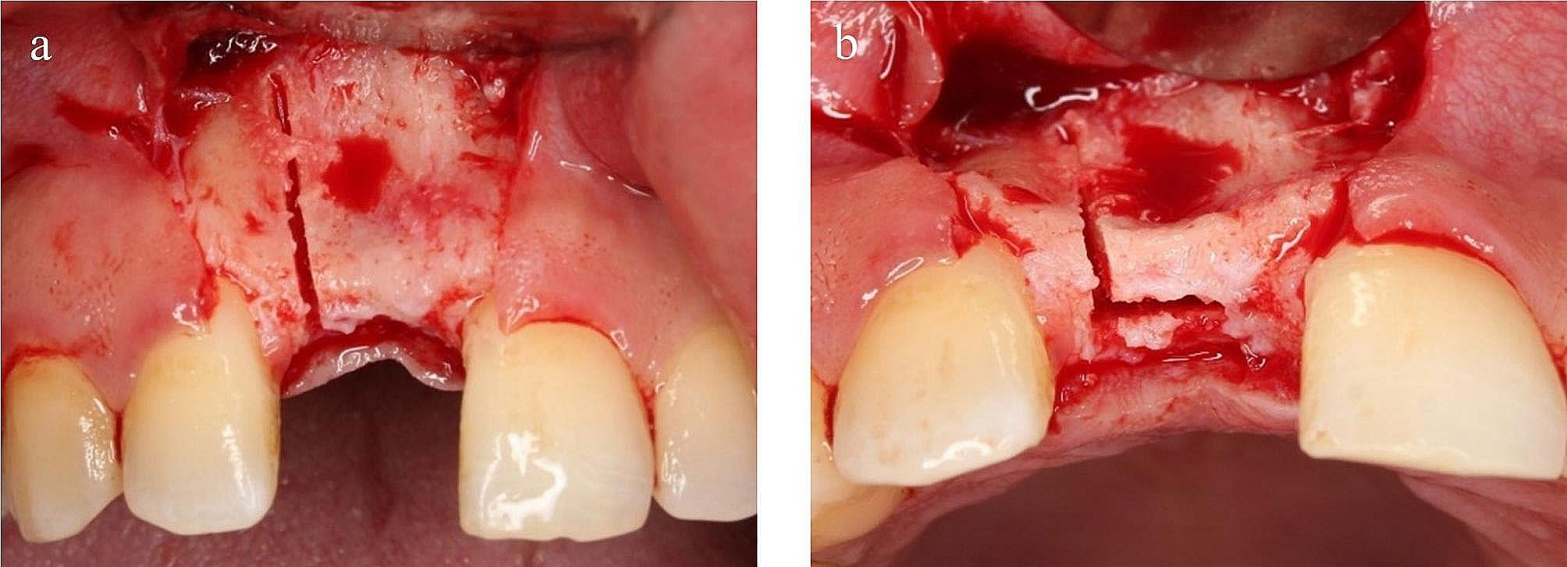
Showing crestal and vertical osteotomies, (**a**) labial view, (**b**) occlusal view

The horizontal osteotomy was propagated apically into the cortical bone by the same piezosurgical tip to a depth matching the length of the implant. Vertical buccal osteotomy/osteotomies were started at the mesial, distal, or mesial and distal ends of the performed crestal osteotomy. Vertical osteotomy/osteotomies were extended apically to meet the crestal osteotomy along its entire depth ensuring complete separation and mobility of the facial bony plate (Fig. 3). In cases where one implant was to be placed, the choice of one vertical osteotomy was made to reduce the possibility of buccal bony plate fracture.

**Fig. 4:**
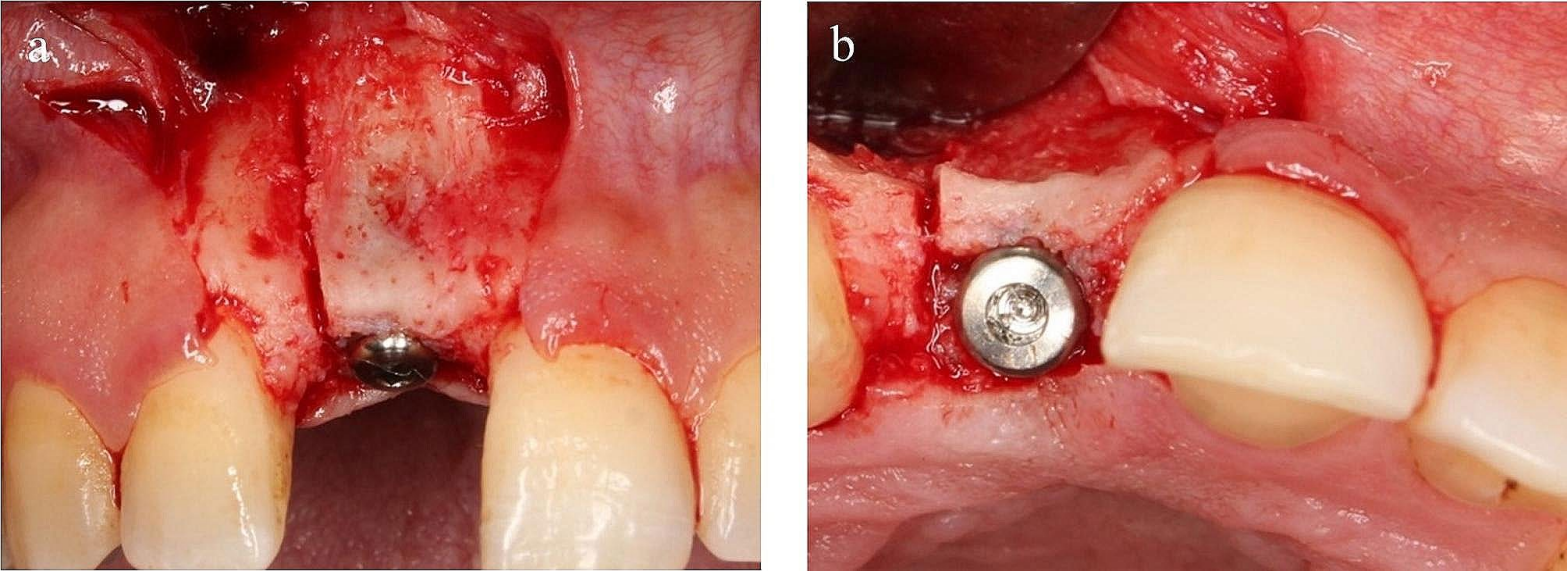
Implant placement flushed with crestal bone and cover screw installed, (**a**) labial view, (**b**) occlusal view

Implant site preparation with the OsteoCare ultra 3.25 drill was performed reaching the assigned implant length. OsteoCare Maxi-Z 2-piece tapered self-drilling self-tapping implant(s)^6^ were placed using a rachet wrench attached to the 2.2 mm ex-driver. Implants were inserted with their platform flushing with the ridge crest then cover screws were placed (Fig. 4). Same size implants (3.75 mm in width and 13 mm in length) were used for all participants.

**Fig. 5:**
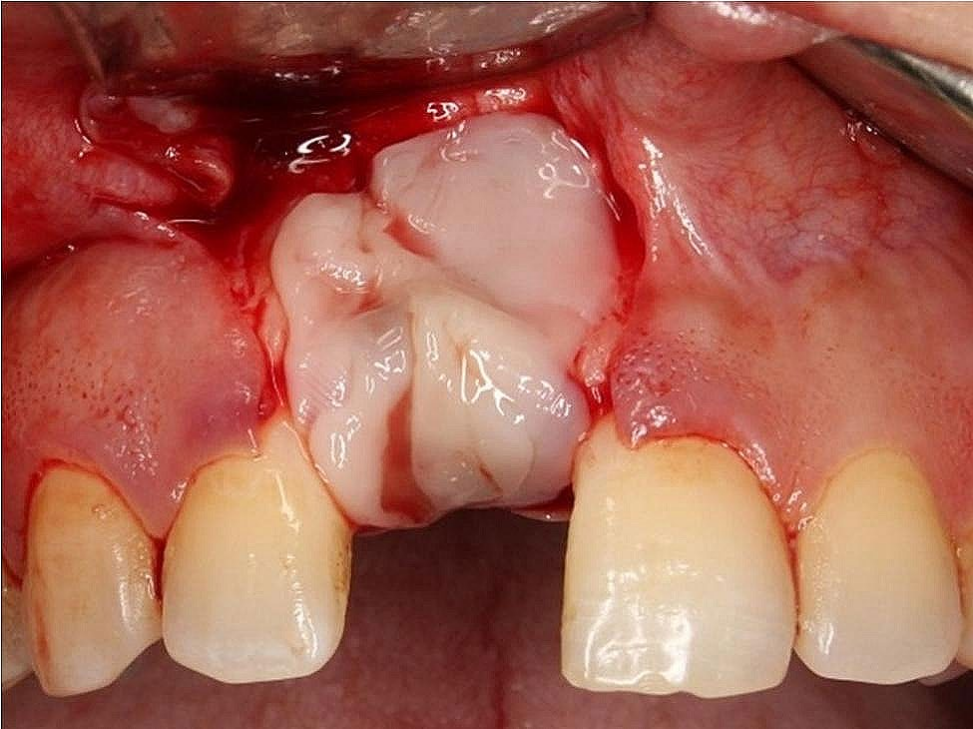
The control group; the surgical site is covered with PRF membrane

**Fig. 6:**
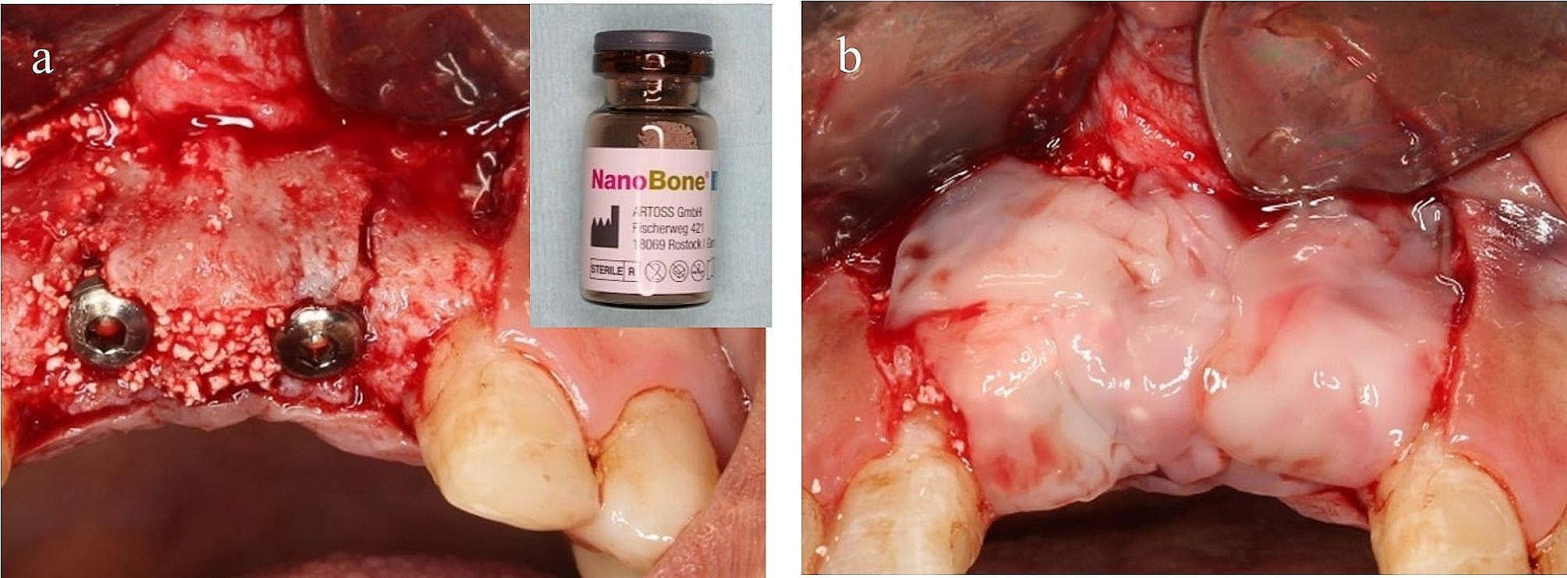
The test group **(a)** Split-site filled with Nanobone^®^, **(b)** Whole surgical site covered with PRF membrane

**Fig. 7:**
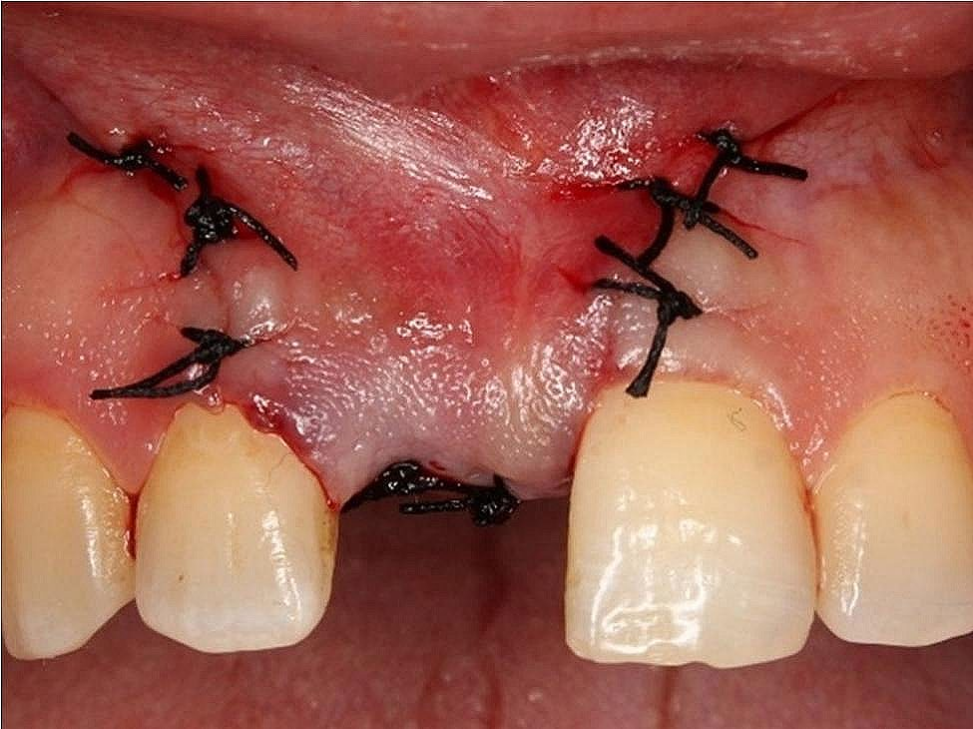
Wound closure with interrupted sutures

For the control group, the surgical site was covered with the PRF membrane only (Fig. 5). For the test group, Nanobone^®^^7^ was mixed with saline and was placed into the splitted site then the surgical site was covered with PRF membrane as same as in the control group (Fig. 6). For both groups periosteal-releasing incision was used for buccal flap advancement to allow for tension-free primary closure then a 4 − 0 silk suture^8^ was used in an interrupted manner to close the flap (Fig. 7).

### Post-surgical protocol

Patients were prescribed cryotherapy immediately after the surgery for one hour. Patients were instructed to monitor and record pain and swelling in the given forms every day immediately after waking up for 7 days.

#### Follow-up (T1)

After seven days radiographic CBCT scans were performed, pain and swelling scale forms were collected, and sutures removal was performed.

#### Prosthetic phase (T2)

After five months radiographic CBCT scan and implant exposure procedures were performed. Healing collars were inserted for one week followed by the placement of permanent abutments, impressions, and fabrication of final restoration.

#### CBCTF process

CBCT scan data at T0, T1, and T2 were collected and imported to the Ondemand3D software for the fusion process. For horizontal bone changes, the fusion process was performed by merging CBCT scan data performed at T0 and T2, while for crestal bone changes, the fusion process was between T1 scans and T2 scans.

**Fig. 8:**
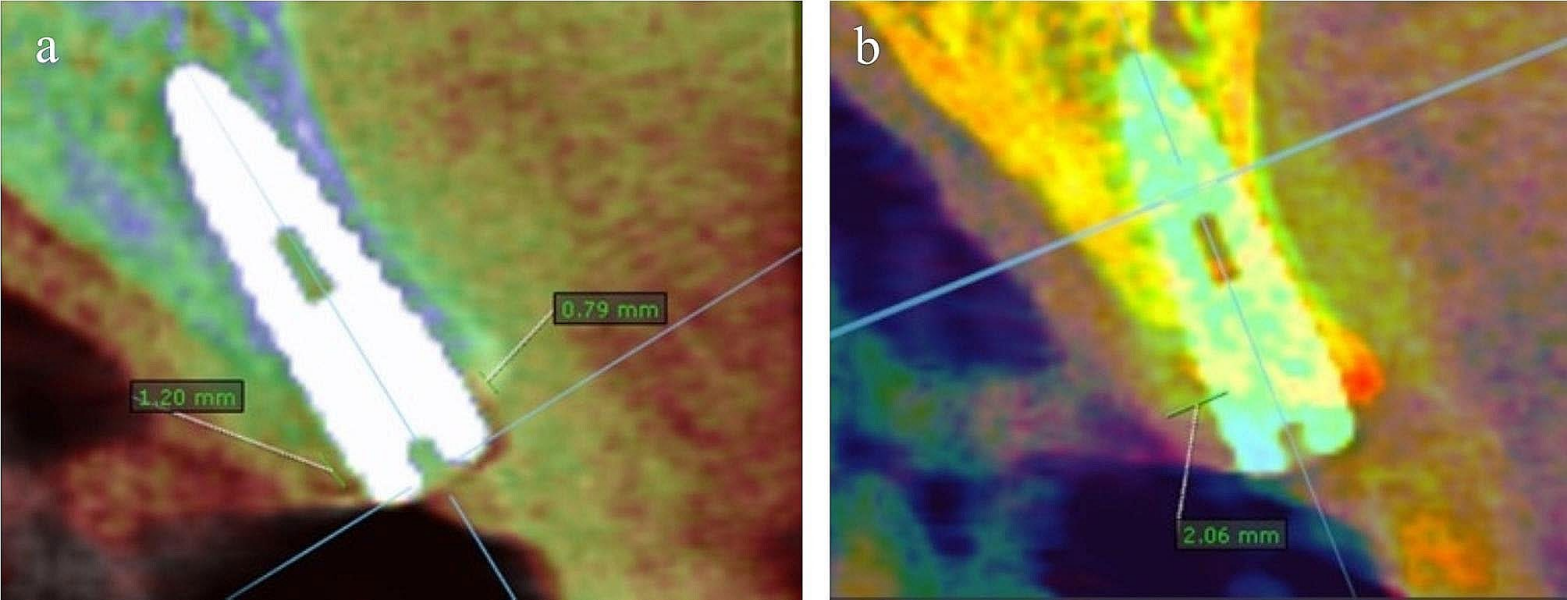
(**a**) Cross-sectional view of CBCTF at T1 (green color) and T2 (blue color) showing the difference in buccal and lingual crestal bone level, (**b**) Cross-sectional view of CBCTF at T0 (yellow-red color) and T2 (green color) showing the difference in horizontal bone width

The two images at the two different time points were overlapped and exactly matched by the software algorithm and presented with different colors for each image. Buccal and lingual crestal bone changes were measured as the difference between T2 and T1 images (Fig. 8 (a)). Horizontal bone changes were calculated by measuring the difference between T0 and T2 images (Fig. 8 (b)).

### Data collection and management

Data-related outcomes were collected pre-surgically and post-surgically in a specific data collection chart for each participant which also contained the participant demographic data and any intra-surgical or post-surgical complications. A file for each participant included the data collection chart, the CBCT scans, and a signed copy of the written informed consent.

### Statistical analysis

Data presented as mean, standard deviation (SD), 95% confidence interval (CI), frequency (n), and percentage (%) when appropriate. Data was explored for normality using Kolmogorov-Smirnov and Shapiro-Wilk tests. Buccal Crestal bone level (mm), lingual crestal bone level (mm), pain, and swelling data showed non-normal distribution, Mann Whitney test was used to compare between study groups. The Wilcoxon signed-rank test was used to compare between follow-up periods.

For Horizontal Bone width (mm), data showed normal distribution, so repeated measures ANOVA was used to compare between study groups and follow-up periods followed by multiple comparisons with Bonferroni adjustment. The significance level was set at *P* ≤ 0.05. Statistical analysis was performed with IBM SPSS Statistics for Windows, Version 26.0. Armonk, NY: IBM Corp.

## Results

### Study patients

Forty medically free patients (16 males, 24 females) were enrolled in the study and randomized, 20 to the test group (9 males, 11 females) and 20 to the control group (7 males, 13 females), and were treated according to the allocated intervention. Healing in all patients was uneventful. Minimal partial soft tissue exposure of single implant cover screw was noticed in a patient in the control group on the palatal side with no effect on healing or osseointegration of the placed implant.

43 implants in total were placed (control group: 22, and test group: 21). Data from one implant in the test group was excluded from statistical analysis and considered a dropout due to poor position. No implant loss was recorded, and all implants achieved successful osseointegration and received final permanent restorations. No cracks, fenestrations, or fractures of the bony plates occurred during splitting, drilling, or implant insertion.

There was no significant statistical difference between both study groups regarding age (test group 35.05 ± 8.61 years, control group 34.50 ± 10.47 years (Table 1)) or gender (test group: 13 females and 7 males, control group: 11 females 11 and 9 males (Tabel 2)).

**Table 1:** Mean and SD of age for study groups

	Group A (Control)		Group B (Nanobone)		*p*-value
	Mean	SD	Mean	SD	
Age	34.50	10.47	35.05	8.61	0.857 NS

NS = Non-significant, *= significant

**Table 2:** Frequency (n) and percentage (%) of gender distribution in study groups

	Group A (Control)		Group B (Nanobone)		p-value
	n	%	n	%	
Female	11	55.0%	13	65.0%	0.519 NS
Male	9	45.0%	7	35.0%	

NS = Non-significant, *= significant

#### Clinical outcomes

##### Change in the buccal crestal bone level

**Fig. 9:**
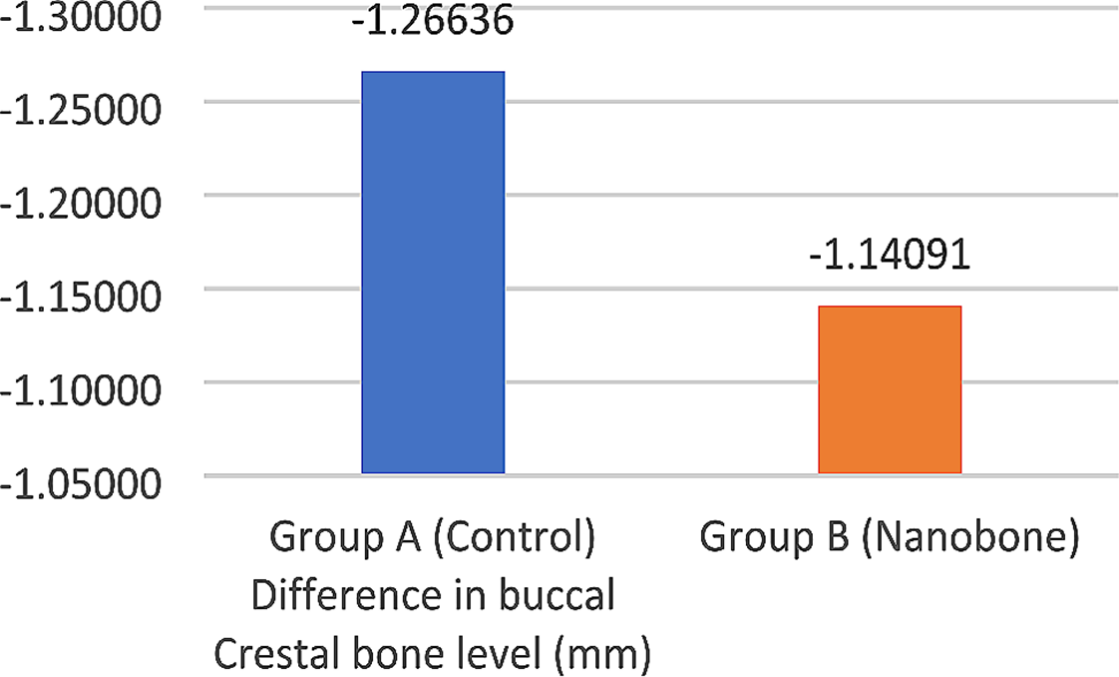
Bar chart showing the mean difference in buccal crestal bone level (mm) for study groups after five months T2

Both groups showed a statistically significant decrease in buccal crestal bone level between T1 and T2 (*p* < 0.001). The mean buccal crestal bone level loss after five months of implant placement was 1.14 ± 0.63 mm for the test group, and 1.26 ± 0.58 mm for the control group. However, there was no significant statistical difference between the study groups at T2 (*p* = 0.549) (Table 3) (Fig. 9).

**Table 3:** Mean, standard deviation (SD), and 95% confidence interval for the difference in buccal crestal bone level (mm) within and between study groups

Buccal crestal bone level (mm)		Paired differences		95% Confidence interval of the difference		*p*-value
		Mean	SD	Lower	Upper	
Within each group significance	Group A (Control)	-1.26636	0.58602	-1.52619	-1.00654	< 0.001*
	Group B (Nanobone)	-1.14091	0.63936	-1.42439	-0.85743	< 0.001*
Between study groups significance						0.549 NS

NS = Non-significant, *= significant

##### Change in the lingual crestal bone level

**Fig. 10:**
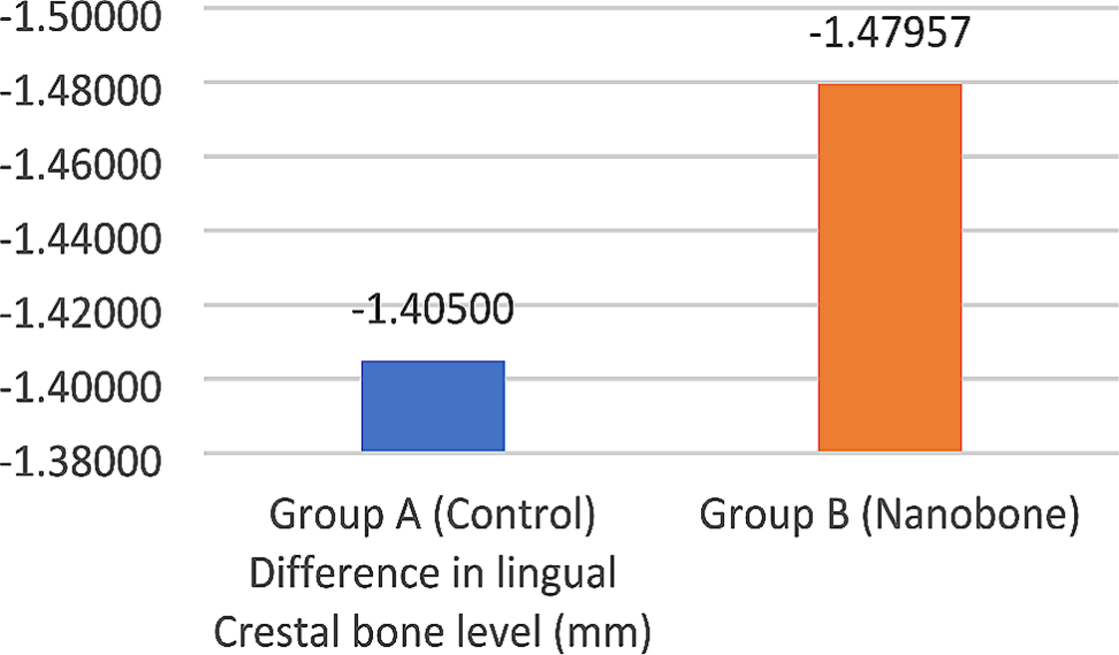
Bar chart showing the mean difference in lingual crestal bone level (mm) for study groups after five months T2

Both groups showed a statistically significant decrease in lingual crestal bone level between T1 and T2 (*p* < 0.001). The mean lingual crestal bone level loss after five months of implant placement was 1.47 ± 0.68 mm for the test group, and 1.40 ± 0.66 mm for the control group. However, there was no significant statistical difference between the study groups at T2 (*p* = 0.674) (Table 4) (Fig. 10).

**Table 4:** Mean, standard deviation (SD), and 95% confidence interval for the difference in lingual crestal bone level (mm) within and between study groups

Lingual crestal bone level (mm)		Paired differences		95% Confidence interval of the difference		*p*-value
		Mean	SD	Lower	Upper	
Within each group significance	Group A (Control)	-1.40500	0.66894	-1.70159	-1.10841	< 0.001*
	Group B (Nanobone)	-1.47957	0.68366	-1.77520	-1.18393	< 0.001*
Between study groups significance						*p* = 0.674 NS

NS = Non-significant, *= significant

##### Change in horizontal bone width

**Fig. 11:**
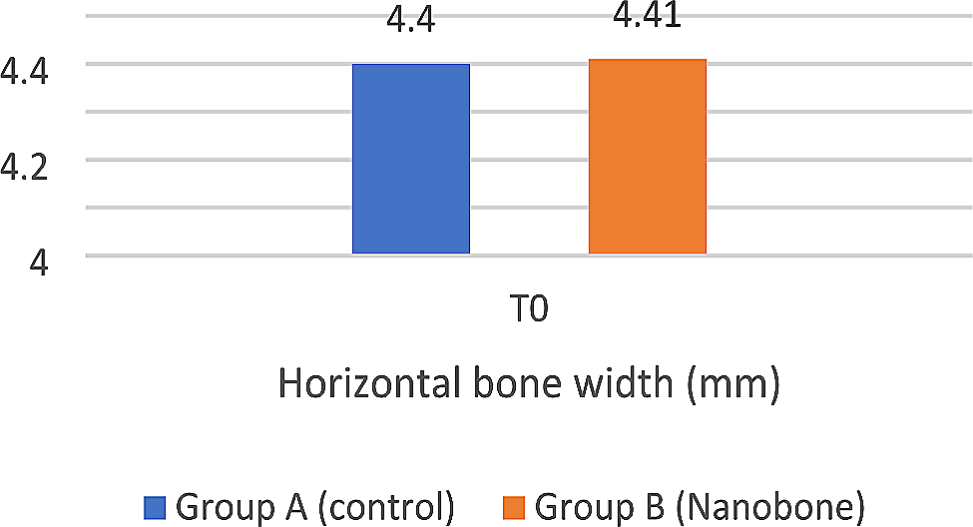
Bar chart showing the mean horizontal bone width (mm) for study groups at baseline T0

At baseline T0, the mean pre-operative alveolar ridge width was 4.41 ± 0.64 mm for the test group and 4.40 ± 0.67 mm for the control group with no significant statistical difference between the two groups (*p* = 0.956) (Table 5) (Fig. 11).

**Table 5:** Mean, standard deviation (SD), and 95% confidence interval for horizontal bone width (mm) for study groups at baseline T0

	Group A (Control)				Group B (Nanobone)				*p*-value
	Mean	SD	95.0% Lower CL	95.0% Upper CL	Mean	SD	95.0% Lower CL	95.0% Upper CL	
Horizontal Bone width (mm)	4.40	0.67	4.11	4.70	4.41	0.64	4.14	4.69	0.956 NS

NS = Non-significant, *= significant

**Fig. 12:**
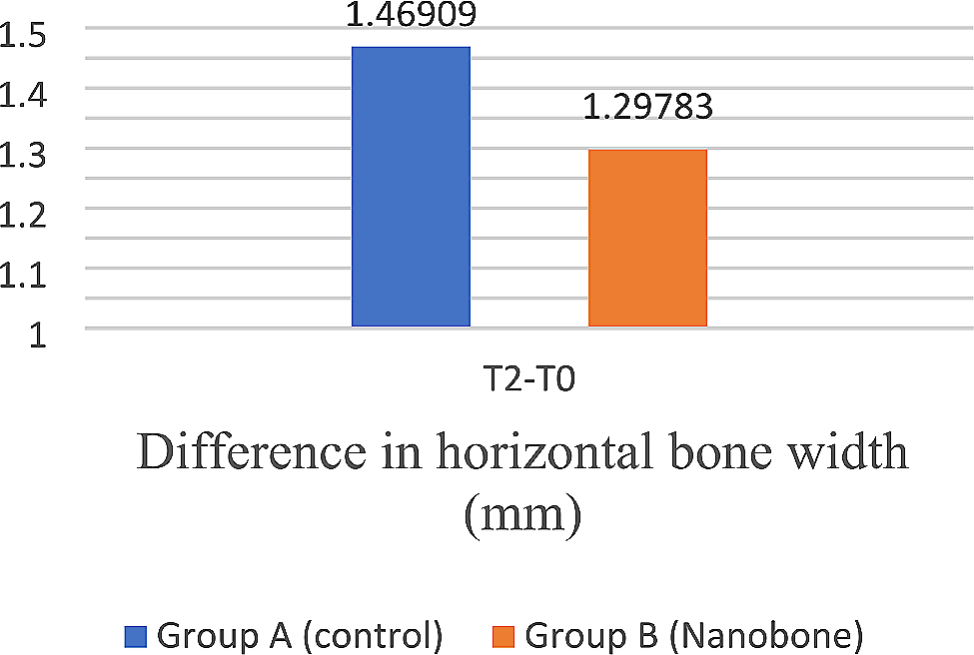
Bar chart showing the mean difference in horizontal bone width (mm) for study groups at T2

All splitted bony ridges achieved sufficient horizontal bone gain to accommodate standard-size implants. The mean alveolar ridge width gain at T2 was 1.29 ± 0.73 mm for the test group and 1.46 ± 0.44 mm for the control group which was statistically significant for both groups (*p* < 0.001) with no significant statistical difference between the study groups at T2 (*p* = 0.231) (Table 6) (Fig. 12).

**Table 6:** Mean, standard deviation (SD), and 95% confidence interval for the difference in horizontal bone width (mm) within and between study groups at T2

Horizontal bone width (mm)			Paired differences		95% Confidence interval of the difference		*p*-value
			Mean	SD	Lower	Upper	
Within study groups	Group A (Control)	T2 - T0	1.46909	0.44872	1.27014	1.66804	< 0.001*
	Group B (Nanobone)	T2 - T0	1.29783	0.73950	0.97804	1.61761	< 0.001*
Between study groups							0.231 NS

NS = Non-significant, *= significant

##### Pain

**Fig. 13:**
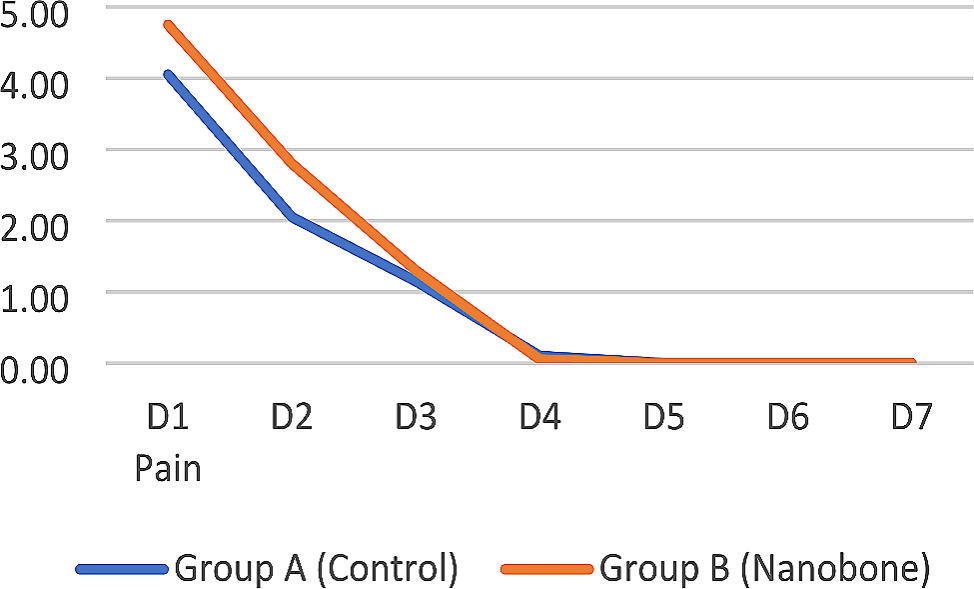
Line chart showing the mean pain reduction for study groups

Regarding post-operative pain, there was no significant statistical difference between both study groups except on day two in the test group which showed slightly higher values. Both study groups exhibited a significant decrease in pain values on the fourth day postoperatively (Fig. 13).

##### Swelling

**Fig. 14:**
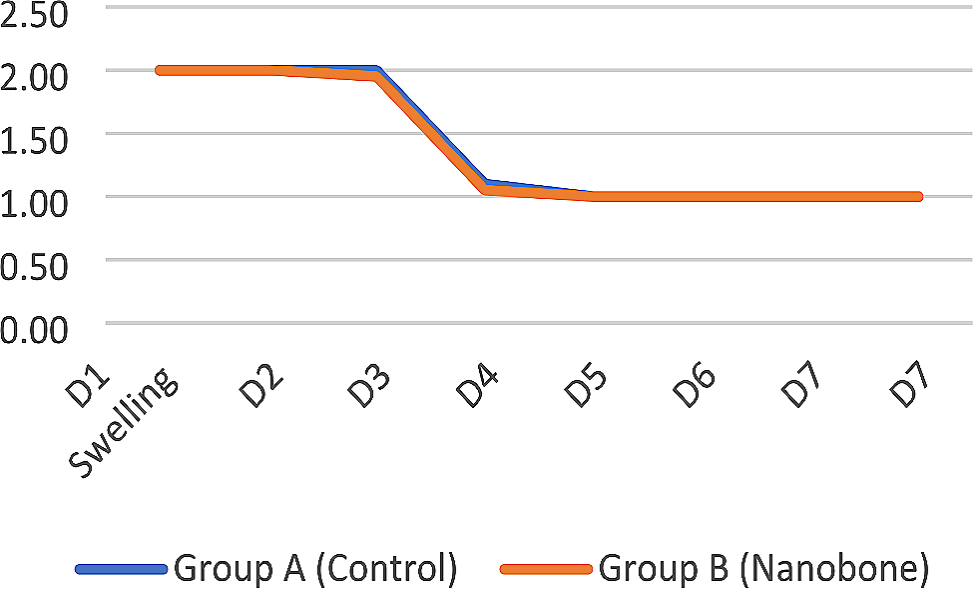
Line chart showing the mean swelling reduction for the tested groups

There was no significant statistical difference regarding swelling between both study groups. Both groups showed a significant decrease in swelling values on the fourth day postoperatively (Fig. 14).

## Discussion

Since its introduction by Dr. Hilt Tatum in the 1970s, the split-crest technique has been used predictably with remarkable success in conjunction with dental implant placement. Split-crest technique for implant placement provides an instant increase in ridge width enabling the insertion of standard-size implants in narrow ridges [20].

The split-crest technique has been known and used for more than fifty years. There are few well-performed trials with high-quality evidence that can be found on the technique in the literature at large, and fewer studies on split-crest using piezosurgical devices in specific.

To the author’s best knowledge and despite the debate present whether to graft the splitted space or not, no randomized controlled trials (RCTs) investigated bone fill and/or crestal bone changes in split-crest using piezosurgical devices comparing grafted versus non-grafted split space in humans to assess and provide accurate data in this specific surgical situation. That provided a strong reason to perform the current study in a path to create conclusive evidence - along with similar studies - about utilizing bone substitutes in split-crest technique.

This study was designed as RCT to provide the highest quality evidence that can be achieved. Random allocation of treatment interventions between the study groups ensured that the estimated effect of the treatment intervention did not deviate from its true value (eliminating the risk of selection bias). The main operator (MW) was blinded at the post-operative data recording phase and statistical analysis generation, thus eliminating the risk of confirmation bias. Post-operative data collection was performed by (RA) which was blinded regarding which patient received which intervention.

The use of piezosurgical devices to perform splitting of the crest is preferred over conventional methods. Results from a systematic review conducted by Godoy-Reina et al. stated that the split-crest technique performed with piezosurgical devices has advantages over drilling or cutting with rotary or conventional instruments. Studies included in the systematic review explained the improved bone healing in relation to the minimal invasive characteristic of the piezosurgical devices which resulted in less inflammation during implant osseointegration. They also reported higher implant stability values after three months compared to the use of other instruments [21]. All patients participated in this study had a good tolerance for the procedure. Healing in all cases was uneventful with no post-operative complications reported. Post-operative pain and swelling were reported to be mild in most cases and moderate in a few of them with no major discomfort. Pain and swelling subsided completely by the fourth day in all cases.

After determining alveolar ridge dimensions with a preoperative CBCT, the procedure started exposure of the remaining alveolar ridge using a full mucoperiosteal flap with two vertical releasing incisions to ensure better visibility during splitting and to allow PRF membrane placement. In this study, the choice was made to make a palatally positioned sub-crestal incision to expose the surgical site. to ensure -at closure- the complete coverage of the splitting site and implant placed as well as any bone substitutes and barrier membranes used.

As a standard procedure, a horizontal osteotomy is performed at the center of the alveolar ridge which is deepened vertically to split the palatal and facial bony plates. There are a few points to be taken into consideration when performing the crestal osteotomy: (1) the vertical depth at which the crestal osteotomy will be terminated, (2) the mesio-distal extension of the osteotomy in the presence of neighboring teeth, and (3) the extension of the osteotomy after the last implant.

The crestal osteotomy is propagated apically to allow easy expansion of the bone and decrease the possibility of baseline fracture. The depth of the crestal osteotomy is variable throughout the literature. It can be terminated short of the planned implant length by three to four millimeters with the rationale of insertion of three to four millimeters of the implant in un-splitted bone to improve primary stability [22–24]. Sammartino et al. reported six millimeters depth for 11.5 mm implant insertion leaving 5.5 mm un-splitted [25].

Another opinion suggests that the depth should be extended beyond the planned implant length allowing a hinge movement at the base of the splitted alveolar crest to facilitate the expansion process [26]. Simion et al. suggested the minimum vertical bone length needed to perform split-crest should be greater than 10 millimeters and the crestal osteotomy depth should extend five to seven millimeters [23]. There is no recommended length for the depth of the crestal osteotomy in relation to implant length throughout the literature.

Reviewing the literature revealed that the choice of vertical depth of the crestal osteotomy is based on clinician choice rather than on standard guidelines. Furthermore, studies utilizing various depths reported implants’ long-term success and stability. No studies compare implant-related outcomes such as stability, success, and survival rates for implants placed in alveolar ridges splitted into different depths. In our study, the choice was to make the depth of the split the same as the length of the placed implant.

The safety margin between the end of the osteotomy and the neighboring teeth should be considered. Some studies reported a one mm safety margin while others reported two mm with no evidence of the reason for choosing a certain distance as a safety margin over the other [22, 25, 27]. No studies investigated or compared the effect of the different safety margins. Safety margin determination will determine the minimum mesio-distal distance between teeth needed to perform splitting. For example, if a two mm safety margin is adopted, the minimum mesio-distal width needed to perform the splitting should not be less than nine millimeters between teeth. While if just a one mm safety margin is to be implemented, the minimum needed mesio-distal distance will be seven millimeters. In this study, a safety margin of two mm was adopted, and a minimal mesio-distal distance of nine mm was implemented to insert a standard implant of 3.75 mm. Precise determination of the safety margin can be crucial in some clinical situations, especially in narrow mesio-distal dimensions bordered by neighboring tooth/teeth.

The core principle of alveolar ridge splitting is to take advantage of bone elasticity which permits separation and widening of the splitted bony plates. Reviewing literature related to alveolar ridge splitting showed that widening\ expansion of the alveolar ridge after the first crestal horizontal osteotomy to accommodate the insertion of adequate size implant can be achieved by: (1) using bone expanders of increasing diameter starting from the narrowest osteotome to the widest [24, 26], (2) making vertical osteotomies at the end of the crestal osteotomy to permit the movement of the separated facial bony plate [23, 26, 28, 29], (3) combination of both [4], (4) inserting tapered dental implants into the split space achieving implant seating and ridge expansion in one step [30]. There are no clear guidelines or limitations for any of the previous procedures in the literature.

In our study, the choice was made to use the tapered dental implants for expansion and to decrease the probability of labial plate fracture, one vertical osteotomy either mesial or distal to the crestal osteotomy was performed in narrow mesio-distal edentulous spaces while two vertical osteotomies were performed at the mesial and distal ends in larger edentulous spaces.

Bone fill properties of NanoBone^®^ were tested in socket preservation in comparison to normal healing after teeth extraction in a split-mouth design study for patients who needed bilateral premolar extraction for orthodontic treatment [31]. Study outcomes compared the difference in gingival invaginations incidence and degree. Results stated partial improvement in gingival invagination degree in sockets grafted with NanoBone^®^. However, Nanobone^®^ use did not eliminate the incidence of gingival invaginations [31]. Bone density values of newly formed bone measured after sinus lifting procedures using NanoBone^®^ were reported to be superior versus bone formed after sinus lifting using tenting procedures [32].

A randomized controlled study by Hommos et al. conducted on thirty patients who required implant placement in atrophied anterior maxillary region compared immediately placed implants after alveolar ridge splitting (done by bone chisels and osteotomes) with and without nano-hydroxyapatite (HA) bone graft (Neo Active Apatite, GHIMAS, Italy). The results of the study stated that grafting the splitted space with nano HA was better than the use of HA with micro granules, other bone grafting materials, or no grafting in terms of bone formation, rigidity, toughness, dimensional stability, and biocompatibility [33].

However, this study entailed a high risk of bias in multiple aspects: (1) high risk of selection bias as there was no random allocation of intervention between study groups but a random selection of patients were included in the study, (2) high risk of detection bias as there was no blinding for outcome assessors. Furthermore, results were reported with subjective terms rather than numerical data presented as mean and SD values. For those reasons, the data from that study are not conclusive regarding the effect of nano HA on bone changes in the split-crest technique.

Abdelqader Altaweel et al. assessed the clinical and radiological outcomes of Nano HA in combination with PRF and amniotic membrane in grafting mandibular splitted alveolar ridges using a piezosurgical device and simultaneous implant insertion [13]. Study groups were (1) Nano HA alone, (2) Nano HA covered by amniotic membrane and (3) Nano HA mixed with PRF and covered with amniotic membrane. Results reported no statistical difference regarding implant stability quotient (ISQ) values and horizontal bone gain. There was a statistically significant difference in crestal bone resorption with less resorption recorded in the third group followed by the second group while the group that utilized Nano HA alone showed the most crestal bone resorption [13]. The presence of Nano HA in all study groups suggests that differences in crestal bone resorption between groups resulted from the addition of PRF and amniotic membrane rather than the effect of bone graft material. For that reason, the effect of Nano HA on crestal bone changes could not be concluded from Abdelqader Altaweel et al. study. The addition of NanoBone^®^ did not show clinical or statistical advantage regarding crestal bone changes or the amount of horizontal bone gain in our study. These results are consistent with other studies [29, 34].

PRF is a leukocyte and platelet-rich fibrin biomaterial that was introduced by Choukroun et al. in 2001 [35]. The added benefit of using PRF was reported in different surgical situations: (1) the use of PRF as a space-filling material into splitted alveolar ridges alone placed in the created gap compared with no filling at all [22], (2) the mixture of PRF with other bone graft in filling the gap [36] and (3) the use of PRF membrane to cover the whole surgical site [37]. The reason behind PRF used in this study was based on its reported positive effect on crestal bone dimensional changes over time [37].

The periosteal incision was performed in this study to ensure tension-free primary closure over the widened alveolar ridge which in turn promotes wound healing and protects the bone graft and PRF membrane [23, 27].

Implants were left for five months for healing before performing second surgery at which healing abutments were installed in this study. Different healing periods after ridge splitting and implant placement had been reported ranging from three months to six months [20, 23, 28, 29, 34] with no clinical clarification of the chosen period. All implants achieved osseointegration and were prosthetically restored.

The split-crest technique using a piezosurgical device for horizontal ridge augmentation provides a good and predictable treatment modality with a high success rate, less patient morbidity, and less overall cost. The decision to place a bone graft in the splitted space should be based on excellent knowledge of the literature and careful evaluation of the clinical situation, otherwise, the addition of a bone graft could be of no significant clinical value.

One of the limitations of the current study is the lack of clear information about the rationale behind the parameters of certain surgical steps in the literature making the choice for one procedure over another completely governed by the personalized preference of the operator. For example, safety margin determination from the end of the crestal osteotomy to the adjacent teeth in bounded edentulous areas considered while splitting the crest, and the depth of the first horizontal osteotomy in relation to the length of the implant. The presence of clear concise guidelines would help the reproducibility of the study. A short follow-up period is another limitation of the current study. Studies with follow-up periods more than one year after implant loading are recommended.

## Conclusion

Within the limitations of this study, we could conclude that Nanobone^®^ has neither clinical nor statistical significance when used in combination with split-crest technique and simultaneous implant placement regarding buccal and lingual crestal bone resorption, horizontal bone width gain, pain, and swelling.

## Data Availability

The data that support the findings of this study are available from the corresponding author upon reasonable request.

## Footnotes

1 https://www.random.org/sequences/.

2 Sunwell Shengdatong Co., Ltd. Beijing, China.

3 PRF BOX. MCTBIO Co., Ltd. Gyeonggi-do, Korea.

4 ARTINIBSA 40 mg/0.01 mg/ml, Inibsa Dental S.L.U, Barcelona, Spain.

5 VarioSurge NSK, Nakanishi Inc., Tochigi, Japan.

6 OsteoCare™ Dental Implant System, Berkshire, United Kingdom.

7 NanoBone®, ARTOSS GmbH, Rostock, Germany.

8 Assut sutures of Switzerland, Assut medical Sa’rl, Switzerland.
